# Acute Acalculous Cholecystitis Perforation in a Child Non-Surgical Management

**DOI:** 10.4021/gr450w

**Published:** 2012-07-20

**Authors:** Hanan M Alghamdi

**Affiliations:** a King Fahad Specialist Hospital-Dammam, Saudi Arabia; b University of Dammam, Saudi Arabia

**Keywords:** Acute acalculous cholecystitis, Gallbladder perforation, Cholecystostomy tube

## Abstract

Spontaneous gallbladder perforation (SGP) is a rare but fatal complication usually associated with acute calculus cholecystitis. Mostly seen in adult patient and rarely reported in children. We report a rare case of aculcoulus gallbladder perforation in a 15 years old child post kidney transplant managed by percutaneous cholecystostomy tube drainage.

## Introduction

Spontaneous gallbladder perforation is uncommon serious complication of mostly gallbladder stone disease. The incidence is variably reported between 3% and 10% [1]. Only around 5 to 10% of acute cholecystitis is due to acalculous cholecystitis with infrequently reported cases of spontaneous perforation [2, 3].

Acute cholecystitis (uncomplicated) occurs more commonly in females, with a female to male ratio of 2:1, however, GBP is more frequently reported in male patients [4]. Furthermore, it’s rarely reported in children. The timing of GBP can be as early as 2 days from the onset of acute cholecystitis, or after a few weeks [5].

## Case Report

**Figure 1:**
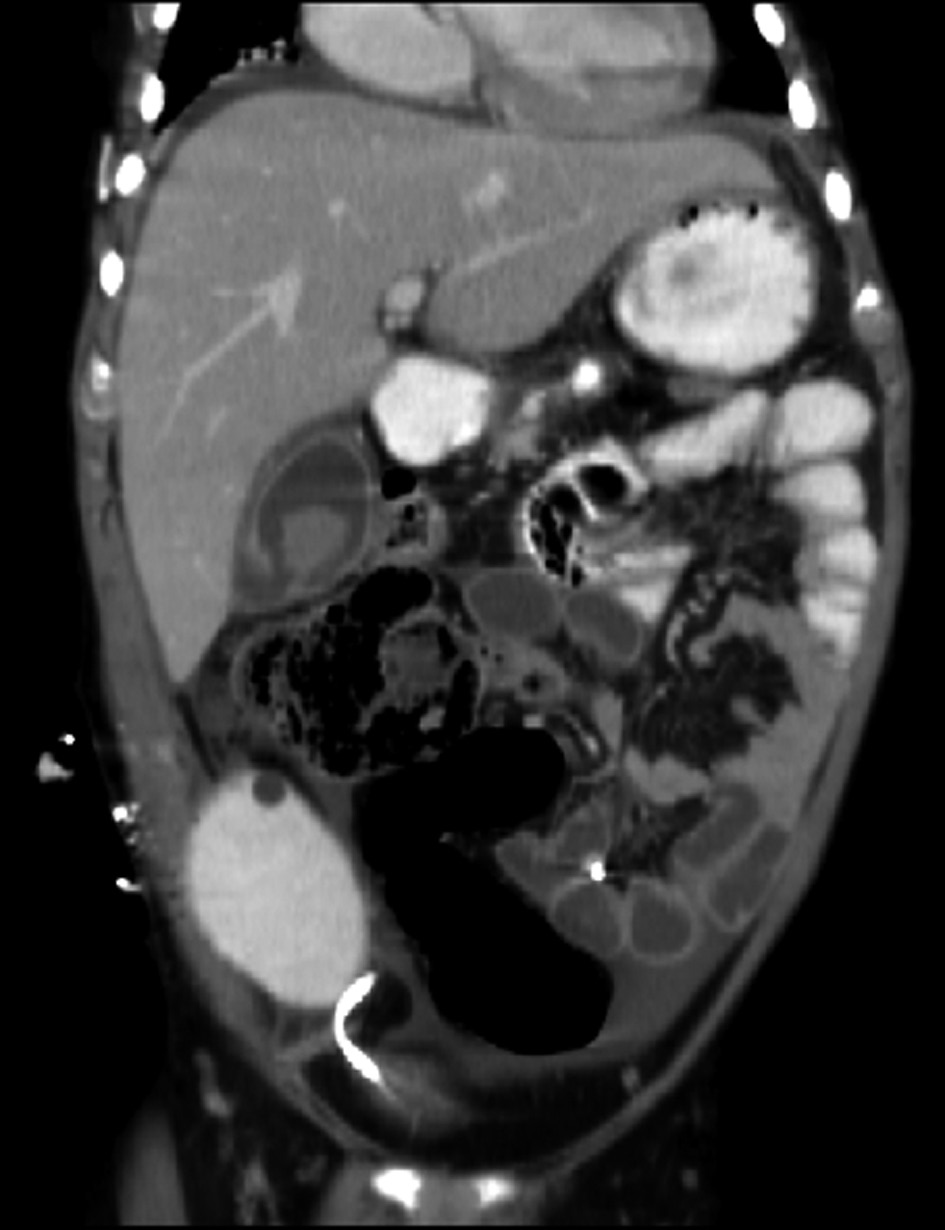
CT scan showing perforated GB: the “HOLE” sign

**Figure 2:**
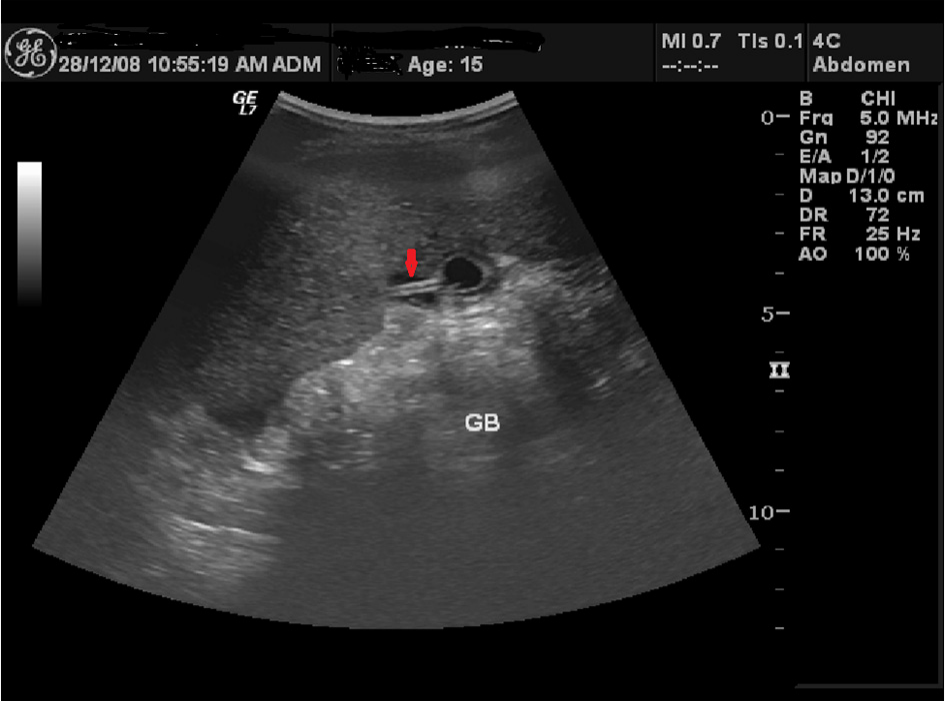
Ultrasound showing the Cholecystostomy tube inside collapsed GB (arrow).

**Figure 3:**
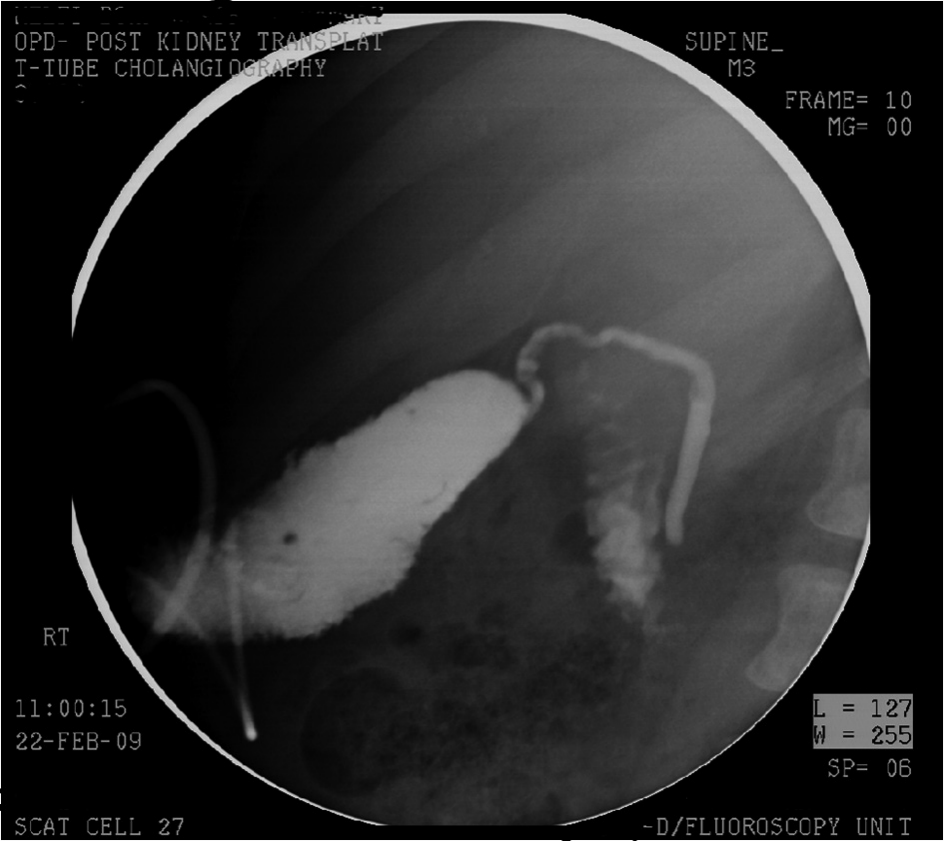
Cholecystostomy tube cholangiogram after 3 month showing no bile leak and well-formed tract.

A 15 years old boy with ESRF due to rapid progressive GN (on peritoneal dialysis) and growth retardation, underwent living related kidney transplantation (from his father), commenced on protocol of triple immunosupression including Tacroluimus, MMF/ Cellcept (750 mg BID) and Prednisolon (to be tapered over three months period to 5 mg) and on bacterial prophylaxis (Bactrim) and CMV prophylaxis (Ganciclovir). On day 22 from the transplant he presented to our emergency department with acute sever colicky right upper abdominal pain and high fever with no similar previous attack. On physical examination he had abdominal distension with generalized tenderness more on the right upper quadrant. His blood work showed leukocytosis (17,000), liver enzyme (including Alkaline phospatase) and renal function were within normal range also CMV antigenemia was negative. Empirically he was started on broad spectrum antibiotics suspecting bacterial peritonitis from the peritoneal dialysis catheter (PDC) and it was connected to gravity drainage (the output fluid was serous). Few hours after admission the abdominal pain start to subside and the PDC drainage became bloody and mixed with bile but the output was decreasing. Then the WBC count went up to 31,000. Repeated ultrasound showed collapsed gallbladder compared to preoperative one with no stones. Further imaging with CT scan confirmed perforated gall bladder (Fig. 1). Under US guidance, a 7F pig tail cholecystostomy catheter and another pig tail tube in the left iliac fossa were inserted. Both tubes were draining biliary fluid (Fig. 2). Patient responded dramatically to this and the pain, fever and leukocytosis subsided gradually over 3 days. One week later the peritoneal pigtail tubes removed after confirming nil output. His Cellcept dose readjusted to 500 mg bid and discharged on the same dose. Three months later the cholecystostomy tube and PDC were removed after confirming no bile leak or collection (Fig. 3). Another follow up imaging after six months showed no recurrence of symptoms and no bile leak. For the next two years the patient never had any recurrence of his symptoms. Accordingly, interval cholecystostomy were not entertained.

## Discussion

In general GBP could be traumatic, iatrogenic or idiopathic (Spontaneous gallbladder perforation). In case of Acalculous Cholecystitis the majority of cases of SGP have been reported in patient with predisposing factors such as in critically ill patients, severe trauma, severe burns, malignancy, infections (e.g. enteric fever), systemic diseases such as diabetes mellitus and atherosclerotic heart disease patient with drugs (e.g. steroids) [6].

The mortality rate is in the range of 11-16% reflects the seriousness of this condition [7, 8].

The most widely used classification of GBP proposed by Niemeier described 3 types, type 1 (acute): associated with generalized biliary peritonitis, type 2 (subacute): consists of localized fluid collection at the site of perforation, pericholecystic abscess and localized peritonitis while in type 3 (chronic): there is an internal or external fistulae formation [9]. Most recent studies have cited higher rates of Type 2 GBP [10].

The widely accepted theory for SGP in acute acalculous cholecystitis suggests gallbladder hypoperfusion (mostly the fundus) as the cause. However, other hypotheses include trauma, congenital abnormality, infection, pancreatic secretions, obstruction and abnormal bile composition [11].

Ultrasonography and computerized tomography may demonstrate abdominal fluid but lack the specificity to diagnose GBP. Significant ultrasound findings of gall bladder thickening (> 3.5 mm), distension, pericholecystic fluid and positive sonographic Murphy sign seen in cases of acute acalculous cholecystitis may also be present in GBP, although none of them is very specific. The most significant radiological finding is the “HOLE” sign, in which the defect in the gall bladder is visualized. The sensitivity of CT in detecting GBP and biliary calculi reported to be 88% and 89%, respectively is higher than those reported for the ultrasound studies. Other modalities used to detect GBP include diagnostic peritoneal lavage, retrograde cholangiography and recently HIDA scan [4].

In the transplant patient SGP is rarely reported in the literature. Mycophenolate mofetil (MMF) is known to cause gastrointestinal dilatation and perforation but rarely reported as a possible cause of gallbladder dilatation and perforation in adult patient. This type of complication never been reported in pediatric transplant recipient [12-14].

Early diagnosis and prompt surgical intervention is pivotal in the management of GBP to decrease the morbidity and mortality associated with this condition. Although, it is difficult to establish the diagnosis of GBP clinically, it is commonly misdiagnosed as a bowel perforation when a patient presents with features suggestive of perforative peritonitis. Cholecystectomy tube and drainage of any abscess or collection, inserted radiological can be a lifesaving and sufficient in the management [15].

In our case we present our non-surgical approach to this rare complication. This approach was prompted by the fact that the gallbladder is not diseased and knowing that surgery could be difficult because of the previous abdominal surgery and the decrease intraabdominal space due to the transplanted adult kidney and being short in the trunk.

Our case is unusual because the patient is a child with no prior history suggestive of gall bladder disease. This patient had a type 1 gallbladder perforation with generalized biliary peritonitis [16].

In conclusion, we suggest that such cases should be properly investigated and underlining cause ascertained. Delay in surgical intervention is the major reason for increased morbidity and mortality associated with GBP. A trail of Cholecystostomy tube in GBP is safe and therapeutic that can avoid a major operative risk in immunosuppressed patient.
